# Finite-time stabilization and *H*∞ control of Port-controlled Hamiltonian systems with disturbances and saturation

**DOI:** 10.1371/journal.pone.0255797

**Published:** 2021-08-16

**Authors:** Baozeng Fu, Qingzhi Wang, Ping Li

**Affiliations:** 1 Institute of Complexity Science, School of Automation, Qingdao University, Qingdao, Shandong Province, China; 2 Shandong Key Laboratory of Industrial Control Technology, Jinan, China; 3 College of Mathematics and Systems Science, Shandong University of Science and Technology, Qingdao, Shandong Province, China; National Huaqiao University, CHINA

## Abstract

The finite-time stabilization and finite-time *H*_∞_ control problems of Port-controlled Hamiltonian (PCH) systems with disturbances and input saturation (IS) are studied in this paper. First, by designing an appropriate output feedback, a strictly dissipative PCH system is obtained and finite-time stabilization result for nominal system is given. Second, with the help of the Hamilton function method and truncation inequality technique, a novel output feedback controller is developed to make the PCH system finite-time stable when IS occurs. Further, a finite-time *H*_∞_ controller is designed to attenuate disturbances for PCH systems with IS, and sufficient conditions are presented. Finally, a numerical example and a circuit example are given to reveal the feasibility of the obtained theoretical results.

## Introduction

The Port-controlled Hamiltonian (PCH) system has been studied well [1–4] since it was put forward [5, 6]. In practical systems, the Hamilton function [7–9], as the total energy containing kinetic energy and potential energy, is a good candidate of Lyapunov function. Apart from the significant Hamilton function, the PCH system’s other structures also have important physical meaning. Thus, many practical systems can be expressed as PCH systems and the PCH system has received wide attention in nonlinear analysis and synthesis [10–13]. Up to now, lots of results on stability analysis and control designs for PCH systems have been presented based on Hamilton function method [14–18].

Under asymptotically stabilized controller, system states can only converge to desired equilibrium points in infinite time, which is a common result. In order to optimize control time, the concept of finite-time stability naturally arises and further finite-time stability theory is developed to improve control performance. In fact, the finite-time control approach has the following significant superiorities: the closed-loop system possesses faster convergence speed and better robustness against uncertainties and disturbances [19–22]. Because of these advantages, finite-time control problems have received a great deal of attention and lots of results have been given in a series of literatures [23–26]. For a class of systems with mismatched disturbances [27], studies the finite-time output regulation problem utilizing the finite-time disturbance observer technique. For a class of nonlinear systems, the finite-time stabilization problem is investigated by constructing a suitable sliding mode control law [29]. For a class of discrete time-delay switched systems, with the help of the average dwell time approach [29]solves the finite-time *H*_∞_ control problem. For a class of Hamiltonian descriptor systems, the finite-time stabilization problem as well as the *H*_∞_ control problem is studied utilizing the Hamilton function method in [30].

Practical systems are often constrained by the limited capacity of physical components. Among these constraint phenomena [31–34], input saturation (IS) is a very common one, which usually destroys the control performance of systems and even results in instability. Since 1950s, control problems of systems with IS has attracted considerable attention and plenty of studies have been reported [35–38]. For uncertain nonstrict-feedback nonlinear systems with IS, the finite-time tracking control problem is addressed via dynamic surface control technique and backstepping approach in [35]. [36] studies finite-time feedback control for input-delay system with nonlinear IS using the comparison function method. Adaptive neural network control for full-state constrained robotic manipulator with IS and time-varying delays is studied in [37]. Utilizing Lyapunov-Krasovskii functional theorem and linear matrix inequalities [38], investigates stabilization problem of time-delay Hamiltonian systems subject to IS. Although lots of results have been presented about stabilization and finite-time stabilization of nonlinear systems in the presence of IS, to our best knowledge, there are fewer results on the finite-time control design of PCH systems with disturbances and IS.

The finite-time stabilization and finite-time *H*_∞_ control problems for PCH systems with disturbances and IS are concerned in this paper. First, the nominal PCH system (without disturbances and IS) is investigated. By utilizing the system’s structural properties and the output feedback strategy, the strictly dissipative PCH system and finite-time stability condition are obtained. Second, the finite-time stabilization problem for the case with IS is studied by designing an output feedback controller using the truncation inequality technique and Hamilton function method. Third, the finite-time *H*_∞_ control problem for the case with IS is addressed, and the sufficient condition is proposed. Finally, a numerical example and a circuit example are given to verify the obtained results.

The contribution is that the strictly dissipative PCH system and finite-time stability condition obtained in this paper, have provided a novel approach to the finite-time stability analysis of PCH systems. Section 3 demonstrates the application of this approach, which discusses the finite-time stabilization and finite-time *H*_∞_ control problems of disturbed PCH systems with IS. We call this novel approach Hamilton function-based analysis method, which includes two cases. (i) The Hamiltonian system is expressed as the strictly dissipative PCH system by designing an appropriate output feedback controller, and the obtained PCH system is finite-time stable. (ii) Under some constraint conditions, the strictly dissipative PCH system cannot be obtained. However, by utilizing the idea of constructing strictly dissipative PCH systems, the positive definite damping matrix can be obtained in the proof, which is very helpful to analyze the finite-time stability of the closed-loop system. It is the advantage that this method applies the systems’ structural properties to controller design and stability analysis effectively. However, different constraints may lead to the theoretical method obtained not being able to solve all related problems. Fortunately, there is at least one broad category where solutions can be found under the theories. We believe that under the PCH systems’ framework, most control problems with saturation can be solved with the development of control theory.

The rest of this article is given below. The problem statement and preliminaries are presented in Section 2. Section 3 addresses the finite-time stabilization problem of the case without and with IS, and the *H*_∞_ control problem of the case with IS and disturbances. Two examples with simulations are presented in Section 4. Section 5 gives the conclusion in final.

**Notation:** The transposition of matrix *g*(*ζ*) is denoted by *g*^T^(*ζ*). The positive definite matrix *R*(*ζ*) is denoted by *R*(*ζ*)>0. The positive semi-definite matrix *R*(*ζ*) is denoted by *R*(*ζ*)≥0. |*a*| stands for the absolute value of real number *a*. ‖*R*‖ represents the Euclidean norm of *R*. ∇*E*(*ζ*) denotes $\frac{\partial E(\zeta )}{\partial \zeta }$.

## Problem statement and preliminaries

A PCH system subject to disturbances and IS is considered

$$\begin{array}{c} \left\{ \begin{array}{c} \dot{\zeta }=\left[J\left(\zeta \right)-R\left(\zeta \right)\right]\nabla E\left(\zeta \right)+g_{1}\left(\zeta \right)sat\left(U\right)+g_{2}\left(\zeta \right)d\left(t\right),\\ y=g_{1}^{\text{T}}\left(\zeta \right)\nabla E\left(\zeta \right),\\ z=M\left(\zeta \right)g_{1}^{\text{T}}\left(\zeta \right)\nabla E\left(\zeta \right) \end{array}\right. \end{array}$$ (1)

where $\zeta \in R^{n}$ is the state, $U\in R^{m}$ is the control input, $y\in R^{m}$ is the output, $d\left(t\right)\in R^{s}$ is the disturbance in *L*_2_, and $z\in R^{q}$ is the penalty signal. The Hamilton function *E*(*ζ*) has a minimum point at *ζ* = 0, and ∇*E*(*ζ*) is the gradient of *E*(*ζ*). $-J^{\text{T}}\left(\zeta \right)=J\left(\zeta \right)\in R^{n\times n}$ is the interconnection matrix, $0\leq R\left(\zeta \right)=R^{\text{T}}\left(\zeta \right)\in R^{n\times n}$ is the damping matrix, *M*(*ζ*) is the weighting matrix, $g_{1}\left(\zeta \right)\in R^{n\times m}$ is the full column rank gain matrix, (*R*, *g*_1_) is a full row rank matrix, $g_{2}\left(\zeta \right)\in R^{n\times s}$, and $sat\left(U\right)={\left[sat\left(U_{1}\right),sat\left(U_{2}\right),\ldots ,sat\left(U_{m}\right)\right]}^{\text{T}}$ is the IS function with

$$\begin{array}{c} sat\left(U_{i}\right)=\{\begin{array}{c} p_{i},\;\;\;\;\;U_{i}>p_{i}>0,\\ U_{i},\;\;\;\;\;p_{i}\geq U_{i}\geq -p_{i},\\ -p_{i},\;\;\;0>-p_{i}>U_{i}, \end{array} \end{array}$$ (2)

*p*_*i*_ is a positive real number which represents the upper bound of the saturated function *sat*(*U*_*i*_).

For the subsequent analysis, the following four lemmas are adopted.

**Lemma 1** ([39]) *Jensen’s inequality*:

$$\begin{array}{c} {\left(\sum_{j=1}^{n}{\left|a_{j}\right|}^{r_{1}}\right)}^{\frac{1}{r_{1}}}\geq {\left(\sum_{j=1}^{n}{\left|a_{j}\right|}^{r_{2}}\right)}^{\frac{1}{r_{2}}},\;0<r_{1}\leq r_{2}, \end{array}$$ (3)

*where r*_1_, *r*_2_ *and a*_*j*_ *are real numbers*.

Let $r_{1}=\frac{1}{c}$ and *r*_2_ = 1 in Lemma 1, then the following inequality is derived

$$\begin{array}{c} {\left(\sum_{j=1}^{n}\left|a_{j}\right|\right)}^{\frac{1}{c}}\leq \sum_{j=1}^{n}{\left|a_{j}\right|}^{\frac{1}{c}},\;c\geq 1. \end{array}$$ (4)

**Lemma 2** ([19]) *The following system is considered*

$$\begin{array}{c} \dot{\psi }=f\left(\psi \right),\;\psi \left(t_{0}\right)=\psi_{0},\;f\left(0\right)=0,\;\psi \in R^{n}. \end{array}$$ (5)

*If there is a C*^1^*radially unbounded Lyapunov function V*(*ψ*) *and a real number b* > 1 *making inequality* (6) *holds along system* (5) *with any* $\psi_{0}\in R^{n}$,

$$\begin{array}{c} \dot{V}\left(\psi \right)\leq -lV^{\frac{1}{b}}\left(\psi \right),\;l>0, \end{array}$$ (6)

*then system* (5) *is globally finite-time stable*.

**Lemma 3** ([32, 40]) *Truncation-inequality technique: Consider the saturation function sat*(*U*) *defined in* (2). *Then, the inequality holds*

$$\begin{array}{c} \delta^{\text{T}}\delta \leq \epsilon U^{\text{T}}U, \end{array}$$ (7)

*where δ* = *sat*(*U*) − *U*, 0 < *ϵ* ≤ 1.

**Lemma 4** ([41]) *The following system is given*

$$\begin{array}{c} \left\{ \begin{array}{c} \dot{\psi }=f\left(\psi \right)+G\left(\psi \right)D\left(t\right),\;f\left(0\right)=0,\\ z=h(\psi ), \end{array}\right. \end{array}$$ (8)

*where ψ is the state*, *z is the penalty signal and D is the disturbance*.

*If there is a function V*(*ψ*) *satisfying the following Hamiltonian-Jacobian inequality*,

$$\begin{array}{c} \nabla^{\text{T}}V\left(\psi \right)f\left(\psi \right)+\frac{1}{2\gamma^{2}}\nabla^{\text{T}}V\left(\psi \right)G\left(\psi \right)G^{\text{T}}\left(\psi \right)\nabla V\left(\psi \right)+\frac{1}{2}h^{\text{T}}\left(\psi \right)h\left(\psi \right)\leq 0, \end{array}$$ (9)

*then the L*_2_*gain from D to z is no bigger thanγ*, *i.e*.,

$$\begin{array}{c} \gamma^{2}\int_{0}^{T}{\left\|D\left(t\right)\right\|}^{2}dt\geq \int_{0}^{T}{\left\|z\left(t\right)\right\|}^{2}dt,\;\forall D\in L_{2}\left[0,T\right], \end{array}$$ (10)

*where V*(*ψ*) > 0 *with ψ* ≠ 0, *V*(0) = 0, *and* *γ* > 0.

**Assumption 1***The Hamilton function E*(*ζ*) *satisfies the condition*$E\left(\zeta \right)=\sum_{i=1}^{n}{\left(\zeta_{i}^{2}\right)}^{\frac{\eta }{2\eta -1}}$, *where η* > 1 *is a real number*.

**Remark 1***As the total energy function of PCH systems, the Hamilton function E*(*ζ*) *is usually selected as the form in Assumption 1, and it represents a very important class of Hamilton functions in mechanical systems*.

In order to deal with the finite-time control problems of PCH system (1) with disturbances and IS, several novel control schemes are presented via output feedback strategies and truncation-inequality technique.

## Main results

In this part, the finite-time control problems of PCH system (1) subject to disturbances and IS are considered. For nominal PCH system, the finite-time stabilization result is given first. Next, the finite-time stabilization result of PCH system is also proposed when IS occurs. Finally, the finite-time *H*_∞_ control problem for the case with disturbances and IS is studied.

### Finite-time stabilization for nominal PCH systems

PCH system (1) with *d*(*t*) = 0 and *sat*(*U*) = *U* is considered in this subsection, i.e., the nominal system (11) is obtained

$$\begin{array}{c} \left\{ \begin{array}{c} \dot{\zeta }=\left[J\left(\zeta \right)-R\left(\zeta \right)\right]\nabla E\left(\zeta \right)+g_{1}\left(\zeta \right)U,\\ y=g_{1}^{\text{T}}\left(\zeta \right)\nabla E\left(\zeta \right). \end{array}\right. \end{array}$$ (11)

For matrix *R*(*ζ*) of system (11), two cases on positive semi-definite and positive definite are first discussed.

Case 1: If *R*(*ζ*) is a positive definite matrix, then system (11) is a strictly dissipative PCH system.
Case 2: If *R*(*ζ*) is a positive semi-definite matrix, an appropriate output feedback controller
  $$\begin{array}{c} U=-Ky \end{array}$$ (12)
  is needed designing to make new matrix $\bar{R}\left(\zeta \right)$ positive definite. Then, system (11) can be transformed into the following strictly dissipative PCH system,
  $$\begin{array}{c} \dot{\zeta }=\left[J\left(\zeta \right)-\bar{R}\left(\zeta \right)\right]\nabla E\left(\zeta \right), \end{array}$$ (13)
  where *K* is a symmetric matrix with proper dimensions,
  $$\begin{array}{c} \bar{R}\left(\zeta \right)=R\left(\zeta \right)+g_{1}\left(\zeta \right)Kg_{1}^{\text{T}}\left(\zeta \right)>0. \end{array}$$ (14)

According to the above discussion, a result is given.

**Theorem 1***Consider nominal PCH system* (11) *with Assumption 1*. *Suppose the condition* (14) *holds, then the output feedback controller* (12) *can finite-time stabilize PCH system* (11).

**Proof.** Substituting output feedback controller (12) into system (11), the strictly dissipative PCH system (13) is derived. Taking *E*(*ζ*) as the Lyapunov function and calculating its derivative along system (11), one obtains

$$\begin{array}{ccc} \dot{E}\left(\zeta \right) & = & \nabla^{\text{T}}E\left(\zeta \right)\left[J\left(\zeta \right)-R\left(\zeta \right)\right]\nabla E\left(\zeta \right)-\nabla^{\text{T}}E\left(\zeta \right)g_{1}\left(\zeta \right)Ky\\ & = & \nabla^{\text{T}}E\left(\zeta \right)\left[J\left(\zeta \right)-R\left(\zeta \right)\right]\nabla E\left(\zeta \right)-\nabla^{\text{T}}E\left(\zeta \right)g_{1}\left(\zeta \right)Kg_{1}^{\text{T}}\left(\zeta \right)\nabla E\left(\zeta \right)\\ & = & \nabla^{\text{T}}E\left(\zeta \right)\left[J\left(\zeta \right)-\bar{R}\left(\zeta \right)\right]\nabla E\left(\zeta \right)\\ & = & -\nabla^{\text{T}}E\left(\zeta \right)\bar{R}\left(\zeta \right)\nabla E\left(\zeta \right)\\ & \leq & -s_{1}\sum_{i=1}^{n}{\left(\frac{2\eta }{2\eta -1}\right)}^{2}\zeta_{i}^{\frac{2}{2\eta -1}}, \end{array}$$ (15)

where

$$\begin{array}{c} s_{1}≔\min_{1\leq i\leq n}\left\{\underset{\zeta \in R^{n}}{\inf}\left\{\sigma_{i}^{\bar{R}}\left(\zeta \right)\right\}\right\}>0, \end{array}$$ (16)

$\sigma_{i}^{\bar{R}}\left(\zeta \right)$ are the eigenvalues of matrix $\bar{R}\left(\zeta \right)$, *i* = 1, 2, …, *n*.

From Lemma 1, it yields

$$\begin{array}{c} \dot{E}\left(\zeta \right)\leq -s_{1}{\left(\frac{2\eta }{2\eta -1}\right)}^{2}\sum_{i=1}^{n}{\left[{\left(\zeta_{i}^{2}\right)}^{\frac{\eta }{2\eta -1}}\right]}^{\frac{1}{\eta }}\leq -s_{1}{\left(\frac{2\eta }{2\eta -1}\right)}^{2}{\left[\sum_{i=1}^{n}{\left(\zeta_{i}^{2}\right)}^{\frac{\eta }{2\eta -1}}\right]}^{\frac{1}{\eta }}, \end{array}$$ (17)

i.e.,

$$\begin{array}{c} \dot{E}\left(\zeta \right)\leq -s_{1}{\left(\frac{2\eta }{2\eta -1}\right)}^{2}E^{\frac{1}{\eta }}\left(\zeta \left(t\right)\right). \end{array}$$ (18)

From Lemma 2, one gets that the closed-loop PCH system (11) with output feedback controller (12) is globally finite-time stable.

**Remark 2***If matrix R*(*ζ*) *is positive definite, i.e., case 1 is considered, PCH system* (11) *is a strictly dissipative PCH system with U* = 0. *In output feedback controller* (12), *we just choose K* = 0, *and the PCH system* (11) *is also globally finite-time stable*.

### Finite-time stabilization for PCH systems with IS

PCH system (1) with *d*(*t*) = 0 is considered in this subsection, i.e.,

$$\begin{array}{c} \left\{ \begin{array}{c} \dot{\zeta }=\left[J\left(\zeta \right)-R\left(\zeta \right)\right]\nabla E\left(\zeta \right)+g_{1}\left(\zeta \right)sat\left(U\right),\\ y=g_{1}^{\text{T}}\left(\zeta \right)\nabla E\left(\zeta \right). \end{array}\right. \end{array}$$ (19)

Via the Hamilton function method and truncation-inequality method, a novel output feedback strategy is developed to solve the finite-time stabilization problem of system (19). Now, the relevant theorem is given as follows.

**Theorem 2***Consider PCH system* (19) *with Assumption 1. If there exists a matrix K with K^T^ = K and proper dimensions satisfying the following condition*,

$$\begin{array}{c} \hat{R}\left(\zeta \right)=R\left(\zeta \right)+g_{1}\left(\zeta \right)Kg_{1}^{\text{T}}\left(\zeta \right)-g_{1}\left(\zeta \right)g_{1}^{\text{T}}\left(\zeta \right)-\epsilon g_{1}\left(\zeta \right)K^{\text{T}}Kg_{1}^{\text{T}}\left(\zeta \right)>0, \end{array}$$ (20)

*then the output feedback controller*

$$\begin{array}{c} U=-Ky, \end{array}$$ (21)

*can finite-time stabilize PCH system* (19) *globally*.

**Proof.** Define *δ* = *sat*(*U*) − *U*, then system (19) can be rewritten as

$$\begin{array}{c} \left\{ \begin{array}{c} \dot{\zeta }=\left[J\left(\zeta \right)-R\left(\zeta \right)\right]\nabla E\left(\zeta \right)+g_{1}\left(\zeta \right)U+g_{1}\left(\zeta \right)\delta ,\\ y=g_{1}^{\text{T}}\left(\zeta \right)\nabla E\left(\zeta \right). \end{array}\right. \end{array}$$ (22)

Choosing Hamilton function *E*(*ζ*) as the Lyapunov function and calculating its derivative, we have

$$\begin{array}{ccc} \dot{E}\left(\zeta \right) & = & \nabla^{\text{T}}E\left(\zeta \right)\left[J\left(\zeta \right)-R\left(\zeta \right)\right]\nabla E\left(\zeta \right)+\nabla^{\text{T}}E\left(\zeta \right)g_{1}\left(\zeta \right)U+\nabla^{\text{T}}E\left(\zeta \right)g_{1}\left(\zeta \right)\delta \\ & = & \nabla^{\text{T}}E\left(\zeta \right)\left[J\left(\zeta \right)-R\left(\zeta \right)\right]\nabla E\left(\zeta \right)-\nabla^{\text{T}}E\left(\zeta \right)g_{1}\left(\zeta \right)Ky+\nabla^{\text{T}}E\left(\zeta \right)g_{1}\left(\zeta \right)\delta \\ & \leq & -\nabla^{\text{T}}E\left(\zeta \right)R\left(\zeta \right)\nabla E\left(\zeta \right)-\nabla^{\text{T}}E\left(\zeta \right)g_{1}\left(\zeta \right)Kg_{1}^{\text{T}}\left(\zeta \right)\nabla E\left(\zeta \right)\\ & & +\nabla^{\text{T}}E\left(\zeta \right)g_{1}\left(\zeta \right)g_{1}^{\text{T}}\left(\zeta \right)\nabla E\left(\zeta \right)+\delta^{\text{T}}\delta \end{array}$$ (23)

Utilizing truncation-inequality technique in Lemma 3, it yields

$$\begin{array}{ccc} \dot{E}\left(\zeta \right) & \leq & -\nabla^{\text{T}}E\left(\zeta \right)\left[R\left(\zeta \right)+g_{1}\left(\zeta \right)Kg_{1}^{\text{T}}\left(\zeta \right)-g_{1}\left(\zeta \right)g_{1}^{\text{T}}\left(\zeta \right)\right]\nabla E\left(\zeta \right)+\epsilon y^{\text{T}}K^{\text{T}}Ky\\ & \leq & -\nabla^{\text{T}}E\left(\zeta \right)\left[R\left(\zeta \right)+g_{1}\left(\zeta \right)Kg_{1}^{\text{T}}\left(\zeta \right)-g_{1}\left(\zeta \right)g_{1}^{\text{T}}\left(\zeta \right)-\epsilon g_{1}\left(\zeta \right)K^{\text{T}}Kg_{1}^{\text{T}}\left(\zeta \right)\right]\nabla E\left(\zeta \right)\\ & = & -\nabla^{\text{T}}E\left(\zeta \right)\hat{R}\left(\zeta \right)\nabla E\left(\zeta \right)\\ & \leq & -s_{2}\sum_{i=1}^{n}{\left(\frac{2\eta }{2\eta -1}\right)}^{2}\zeta_{i}^{\frac{2}{2\eta -1}}, \end{array}$$ (24)

where

$$\begin{array}{c} s_{2}≔\min_{1\leq i\leq n}\left\{\underset{\zeta \in R^{n}}{\inf}\left\{\sigma_{i}^{\hat{R}}\left(\zeta \right)\right\}\right\}>0, \end{array}$$ (25)

$\sigma_{i}^{\hat{R}}\left(\zeta \right)$ are the eigenvalues of matrix $\hat{R}\left(\zeta \right)$, *i* = 1, 2, …, *n*.

According to Lemma 1, one obtains

$$\begin{array}{ccc} \dot{E}\left(\zeta \right) & \leq & -s_{2}{\left(\frac{2\eta }{2\eta -1}\right)}^{2}\sum_{i=1}^{n}{\left[{\left(\zeta_{i}^{2}\right)}^{\frac{\eta }{2\eta -1}}\right]}^{\frac{1}{\eta }}\leq -s_{2}{\left(\frac{2\eta }{2\eta -1}\right)}^{2}{\left[\sum_{i=1}^{n}{\left(\zeta_{i}^{2}\right)}^{\frac{\eta }{2\eta -1}}\right]}^{\frac{1}{\eta }}\\ & = & -s_{2}{\left(\frac{2\eta }{2\eta -1}\right)}^{2}E^{\frac{1}{\eta }}\left(\zeta \left(t\right)\right). \end{array}$$ (26)

According to the above analysis and Lemma 2, it can be concluded output feedback controller (21) can finite-time stabilize PCH system (20).

**Remark 3***Under IS constraints, the strictly dissipative PCH system is not obtained and hence the finite-time stabilization result cannot be yielded directly. However, the positive definite damping matrix is obtained utilizing the idea of constructing strictly dissipative PCH systems and truncation-inequality technique, which helps to prove the finite-time stability of the closed-loop system in Theorem 2*.

### Finite-time *H*_∞_ control for PCH systems with IS

In this subsection, PCH system (1) with *d*(*t*)≠0 is considered. To investigate the finite-time *H*_∞_ control problem, a novel output feedback controller is designed using Hamiltonian-Jacobian inequality technique and truncation-inequality method. And the result is derived as follows.

**Theorem 3***Consider PCH system* (1) *with Assumption 1. Suppose there exists a symmetric matrix K with proper dimensions and a number γ* > 0 *satisfying the following conditions*,

$$\begin{array}{c} \overset{ˇ}{R}\left(\zeta \right)=R\left(\zeta \right)+g_{1}\left(\zeta \right)\left[K-\frac{1}{2}\epsilon {\bar{K}}^{\text{T}}\bar{K}-\frac{1}{2}I_{m}\right]g_{1}^{\text{T}}\left(\zeta \right)>0, \end{array}$$ (27)

$$\begin{array}{c} \tilde{R}\left(\zeta \right)=\overset{ˇ}{R}\left(\zeta \right)+\frac{1}{2\gamma^{2}}\left[g_{1}\left(\zeta \right)g_{1}^{\text{T}}\left(\zeta \right)-g_{2}\left(\zeta \right)g_{2}^{\text{T}}\left(\zeta \right)\right]\geq 0, \end{array}$$ (28)

*then the output feedback controller*

$$\begin{array}{c} U=-\left(K+\frac{1}{2}M^{\text{T}}M+\frac{1}{2\gamma^{2}}I_{m}\right)y=-\bar{K}y \end{array}$$ (29)

*can effectively solve the finite-time H*_∞_ *control problem of PCH system* (1).

**Proof.** Set *δ* = *sat*(*U*) − *U*. Substituting output feedback control law (29) into system (1), the following closed-loop system is obtained

$$\begin{array}{c} \left\{ \begin{array}{c} \dot{\zeta }=\left[J\left(\zeta \right)-\left(R\left(\zeta \right)+g_{1}\left(\zeta \right)\left(K+\frac{1}{2}M^{\text{T}}M+\frac{1}{2\gamma^{2}}I_{m}\right)g_{1}^{\text{T}}\left(\zeta \right)\right)\right]\nabla E\left(\zeta \right)\\ \;\;\;\;\;+g_{1}\left(\zeta \right)\delta +g_{2}\left(\zeta \right)d\left(t\right)≔f\left(\zeta \right)+g_{2}\left(\zeta \right)d\left(t\right),\\ y=g_{1}^{\text{T}}\left(\zeta \right)\nabla E\left(\zeta \right),\\ z=M\left(\zeta \right)g_{1}^{\text{T}}\left(\zeta \right)\nabla E\left(\zeta \right)≔h\left(\zeta \right). \end{array}\right. \end{array}$$ (30)

The proof is divided into two parts. Part 1, system (30) has a finite-time *L*_2_ gain. Part 2, when *d*(*t*) = 0, system (30) is finite-time stable.

We will prove part 1 first. Choose the Lyapunov function *E*(*ζ*), and define

$$\begin{array}{c} W\left(\zeta \right)=\nabla^{\text{T}}E\left(\zeta \right)f\left(\zeta \right)+\frac{1}{2\gamma^{2}}\nabla^{\text{T}}E\left(\zeta \right)g_{2}\left(\zeta \right)g_{2}^{\text{T}}\left(\zeta \right)\nabla E\left(\zeta \right)+\frac{1}{2}h^{\text{T}}\left(\zeta \right)h\left(\zeta \right). \end{array}$$ (31)

According to truncation-inequality and Young’s inequality, one has

$$\begin{array}{ccc} W(\zeta ) & = & -\nabla^{\text{T}}E\left(\zeta \right)\left[R\left(\zeta \right)+g_{1}\left(\zeta \right)\left(K+\frac{1}{2}M^{\text{T}}M+\frac{1}{2\gamma^{2}}I_{m}\right)g_{1}^{\text{T}}\left(\zeta \right)\right]\nabla E\left(\zeta \right)\\ & & +\nabla^{\text{T}}E\left(\zeta \right)g_{1}\left(\zeta \right)\delta +\frac{1}{2\gamma^{2}}\nabla^{\text{T}}E\left(\zeta \right)g_{2}\left(\zeta \right)g_{2}^{\text{T}}\left(\zeta \right)\nabla E\left(\zeta \right)\\ & & +\frac{1}{2}\nabla^{\text{T}}E\left(\zeta \right)g_{1}\left(\zeta \right)M^{\text{T}}Mg_{1}^{\text{T}}\left(\zeta \right)\nabla E\left(\zeta \right)\\ & \leq & -\nabla^{\text{T}}E\left(\zeta \right)\left[R\left(\zeta \right)+g_{1}\left(\zeta \right)\left(K+\frac{1}{2\gamma^{2}}I_{m}\right)g_{1}^{\text{T}}\left(\zeta \right)\right]\nabla E\left(\zeta \right)\\ & & +\frac{1}{2}\nabla^{\text{T}}E\left(\zeta \right)g_{1}\left(\zeta \right)g_{1}^{\text{T}}\left(\zeta \right)\nabla E\left(\zeta \right)\\ & & +\frac{1}{2}\delta^{\text{T}}\delta +\frac{1}{2\gamma^{2}}\nabla^{\text{T}}E\left(\zeta \right)g_{2}\left(\zeta \right)g_{2}^{\text{T}}\left(\zeta \right)\nabla E\left(\zeta \right)\\ & \leq & -\nabla^{\text{T}}E\left(\zeta \right)\left[R\left(\zeta \right)+g_{1}\left(\zeta \right)\left(K+\frac{1}{2\gamma^{2}}I_{m}\right)g_{1}^{\text{T}}\left(\zeta \right)\right]\nabla E\left(\zeta \right)\\ & & +\frac{1}{2}\nabla^{\text{T}}E\left(\zeta \right)g_{1}\left(\zeta \right)g_{1}^{\text{T}}\left(\zeta \right)\nabla E\left(\zeta \right)+\frac{1}{2\gamma^{2}}\nabla^{\text{T}}E\left(\zeta \right)g_{2}\left(\zeta \right)g_{2}^{\text{T}}\left(\zeta \right)\nabla E\left(\zeta \right)\\ & & +\frac{1}{2}\epsilon \nabla^{\text{T}}E\left(\zeta \right)g_{1}\left(\zeta \right){\bar{K}}^{\text{T}}\bar{K}g_{1}^{\text{T}}\left(\zeta \right)\nabla E\left(\zeta \right)\\ & = & -\nabla^{\text{T}}E\left(\zeta \right)[R\left(\zeta \right)+g_{1}\left(\zeta \right)\left(K-\frac{1}{2}\epsilon {\bar{K}}^{\text{T}}\bar{K}-\frac{1}{2}I_{m}\right)g_{1}^{\text{T}}\left(\zeta \right)\\ & & +\frac{1}{2\gamma^{2}}\left(g_{1}\left(\zeta \right)g_{1}^{\text{T}}\left(\zeta \right)-g_{2}\left(\zeta \right)g_{2}^{\text{T}}\left(\zeta \right)\right)]\nabla E\left(\zeta \right)\\ & = & -\nabla^{\text{T}}E\left(\zeta \right)\tilde{R}\left(\zeta \right)E\left(\zeta \right). \end{array}$$ (32)

Based on (32), it follows from condition (28) that

$$\begin{array}{c} W(\zeta )\leq 0. \end{array}$$ (33)

According to the Hamiltonian-Jacobian inequality in Lemma 4, one gets the *L*_2_ gain of system (30) is not bigger than *γ*.

Next, part 2 will be proved. Calculating the derivative of *E*(*ζ*) when *d*(*t*) = 0, one obtains the following result,

$$\begin{array}{ccc} \dot{E}\left(\zeta \right) & = & \nabla^{\text{T}}E\left(\zeta \right)\left[J\left(\zeta \right)-\left(R\left(\zeta \right)+g_{1}\left(\zeta \right)\left(K+\frac{1}{2}M^{\text{T}}M+\frac{1}{2\gamma^{2}}I_{m}\right)g_{1}^{\text{T}}\left(\zeta \right)\right)\right]\nabla E\left(\zeta \right)\\ & & +\nabla^{\text{T}}E\left(\zeta \right)g_{1}\left(\zeta \right)\delta \\ & \leq & -\nabla^{\text{T}}E\left(\zeta \right)\left[R\left(\zeta \right)+g_{1}\left(\zeta \right)\left(K+\frac{1}{2}M^{\text{T}}M+\frac{1}{2\gamma^{2}}I_{m}\right)g_{1}^{\text{T}}\left(\zeta \right)\right]\nabla E\left(\zeta \right)\\ & & +\frac{1}{2}\nabla^{\text{T}}E\left(\zeta \right)g_{1}\left(\zeta \right)g_{1}^{\text{T}}\left(\zeta \right)\nabla E\left(\zeta \right)+\frac{1}{2}\delta^{\text{T}}\delta \\ & \leq & -\nabla^{\text{T}}E\left(\zeta \right)\left[R\left(\zeta \right)+g_{1}\left(\zeta \right)\left(K+\frac{1}{2}M^{\text{T}}M+\frac{1}{2\gamma^{2}}I_{m}\right)g_{1}^{\text{T}}\left(\zeta \right)\right]\nabla E\left(\zeta \right)\\ & & +\frac{1}{2}\nabla^{\text{T}}E\left(\zeta \right)g_{1}\left(\zeta \right)g_{1}^{\text{T}}\left(\zeta \right)\nabla E\left(\zeta \right)+\frac{1}{2}\epsilon U^{\text{T}}U\\ & = & -\nabla^{\text{T}}E\left(\zeta \right)[R\left(\zeta \right)+g_{1}\left(\zeta \right)(K+\frac{1}{2}M^{\text{T}}M\\ & & +\frac{1}{2\gamma^{2}}I_{m}-\frac{1}{2}I_{m}-\frac{1}{2}\epsilon {\bar{K}}^{\text{T}}\bar{K})g_{1}^{\text{T}}\left(\zeta \right)]\nabla E\left(\zeta \right)\\ & = & -\nabla^{\text{T}}E\left(\zeta \right)\left[\overset{ˇ}{R}\left(\zeta \right)+g_{1}\left(\zeta \right)\left(\frac{1}{2}M^{\text{T}}M+\frac{1}{2\gamma^{2}}I_{m}\right)g_{1}^{\text{T}}\left(\zeta \right)\right]\nabla E\left(\zeta \right). \end{array}$$ (34)

Condition (27) implies matrix $\overset{ˇ}{R}\left(\zeta \right)+g_{1}\left(\zeta \right)\left(\frac{1}{2}M^{\text{T}}M+\frac{1}{2\gamma^{2}}I_{m}\right)g_{1}^{\text{T}}\left(\zeta \right)≔\overset{`}{R}\left(\zeta \right)>0$.

Let

$$\begin{array}{c} s_{3}≔\min_{1\leq i\leq n}\left\{\underset{\zeta \in R^{n}}{\inf}\left\{\sigma_{i}^{\overset{`}{R}}\left(\zeta \right)\right\}\right\}>0, \end{array}$$ (35)

where $\sigma_{i}^{\overset{`}{R}}\left(\zeta \right)$ are the eigenvalues of matrix $\overset{`}{R}\left(\zeta \right)$, *i* = 1, 2, …, *n*.

Thus, inequality (34) can be written as

$$\begin{array}{c} \dot{E}\left(\zeta \right)\leq -s_{3}\sum_{i=1}^{n}{\left(\frac{2\eta }{2\eta -1}\right)}^{2}\zeta_{i}^{\frac{2}{2\eta -1}}. \end{array}$$ (36)

From Lemma 1, it is further deduced that

$$\begin{array}{ccc} \dot{E}\left(\zeta \right) & \leq & -s_{3}{\left(\frac{2\eta }{2\eta -1}\right)}^{2}\sum_{i=1}^{n}{\left[{\left(\zeta_{i}^{2}\right)}^{\frac{\eta }{2\eta -1}}\right]}^{\frac{1}{\eta }}\leq -s_{3}{\left(\frac{2\eta }{2\eta -1}\right)}^{2}{\left[\sum_{i=1}^{n}{\left(\zeta_{i}^{2}\right)}^{\frac{\eta }{2\eta -1}}\right]}^{\frac{1}{\eta }}\\ & = & -s_{3}{\left(\frac{2\eta }{2\eta -1}\right)}^{2}E^{\frac{1}{\eta }}\left(\zeta \left(t\right)\right). \end{array}$$ (37)

According to Lemma 2, one gets that when *d*(*t*) = 0, the closed-loop system (30) is globally finite-time stable.

Therefore, the finite-time *H*_∞_ control problem of PCH system (1) is solved.

**Remark 4***In this paper, the salient features are reflected in the following several aspects compared with the existing research results* [30, 31, 35–38]. *(i) Compared with the inequality technique in* [31, 36], *the truncation inequality technique, borrowed to address IS, is more feasible. (ii) Different from the the result that the state is bounded in finite-time* [35], *our aim is that the state converges to the equilibrium point in finite-time. (iii) Via the Hamilton function method and finite-time control technique, two novel output feedback control laws are designed to solve the finite-time stabilization and finite-time H*_∞_ *control problems of PCH systems in the presence of disturbances and IS, which is different from the stabilization of Hamiltonian systems without disturbances and with IS* [38] *and the finite-time H*_∞_ *control for Hamiltonian descriptor systems without IS* [30].

## Simulations

This section presents two simulation examples to reveal the feasibility of the proposed control methods.

**Example 1.** The following PCH system with IS is considered

$$\begin{array}{c} \left\{ \begin{array}{c} \dot{x}=\left[J\left(x\right)-R\left(x\right)\right]\nabla E\left(x\right)+g_{1}\left(x\right)sat\left(u\right),\\ y=g_{1}^{\text{T}}\left(x\right)\nabla E\left(x\right), \end{array}\right. \end{array}$$ (38)

where the state $x\in R^{2}$, the Hamilton function $E\left(x\right)=x_{1}^{\frac{4}{3}}+x_{2}^{\frac{4}{3}}$, $g_{1}={\left(0,1\right)}^{\text{T}}$, $J=(\begin{array}{cc} 0 & -1\\ 1 & 0 \end{array})$, $R=(\begin{array}{cc} 2 & 0\\ 0 & 0 \end{array})$, sat(u)∈R is the saturated control input and given as

$$\begin{array}{c} sat\left(u\right)=\{\begin{array}{c} 0.2,\;\;\;\;\;u>0.2,\\ u,\;\;\;\;\;0.2\geq u\geq -0.2,\\ -0.2,\;\;\;u<-0.2. \end{array} \end{array}$$ (39)

By choosing *K* = 2 and $∊=\frac{1}{5}$, we know the condition (20) holds, where

$$\begin{array}{c} \hat{R}\left(x\right)=R\left(x\right)+g_{1}\left(x\right)Kg_{1}^{\text{T}}\left(x\right)-g_{1}\left(x\right)g_{1}^{\text{T}}\left(x\right)-\epsilon g_{1}\left(x\right)K^{\text{T}}Kg_{1}^{\text{T}}\left(x\right)=(\begin{array}{cc} 2 & 0\\ 0 & \frac{1}{5} \end{array})>0, \end{array}$$ (40)

and all the conditions hold in Theorem 2.

Based on Eq (21), the output feedback law is designed as

$$\begin{array}{c} u=-Ky=-2y. \end{array}$$ (41)

According to Theorem 2, it is easy to get that control law (41) can finite-time stabilize system (38).

To demonstrate the feasibility of the control strategy, the initial condition *x*_1_(0) = 1 = *x*_2_(0) is first given and further the simulation is carried out.

**Fig 1:**
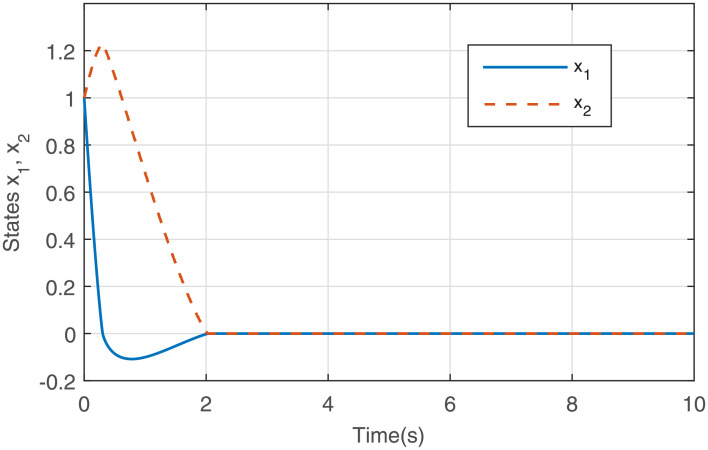
States *x*_1_ and *x*_2_ with *sat*(*u*).

**Fig 2:**
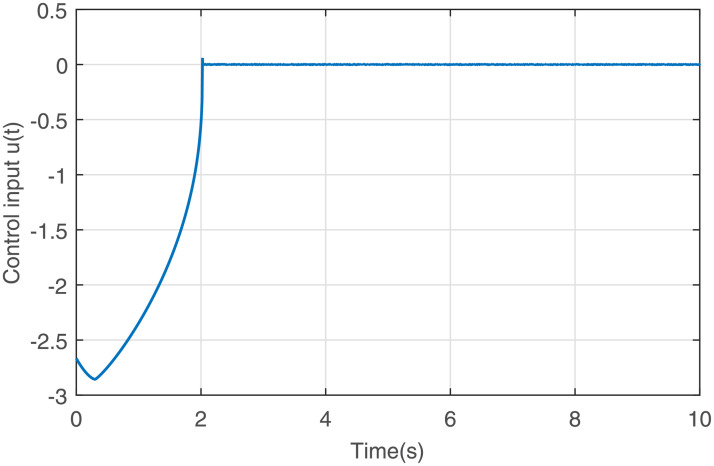
Response curve of the output feedback signal.

**Fig 3:**
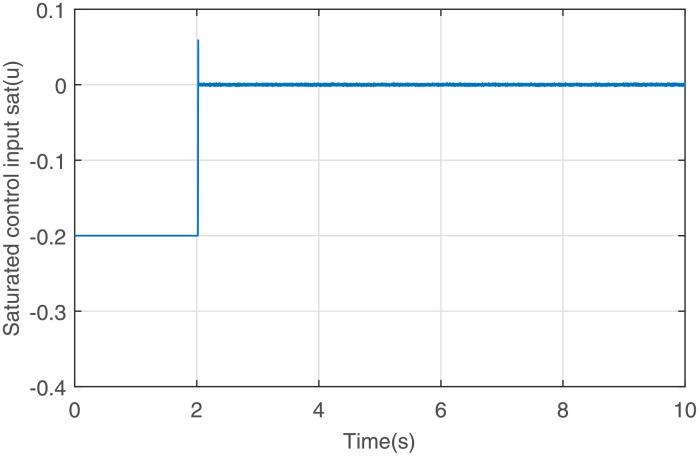
Response curve of the saturated control input *sat*(*u*).

Figs 1 to 3 present the simulation results. Fig 1 illustrates that the states *x*_1_ and *x*_2_ converge to the equilibrium point fast via the output feedback control (41) when IS is considered, i.e., closed-loop system (38) is finite-time stable. The response curve of the output feedback signal is given in Fig 2. Fig 3 is the response curve of the saturated control input *sat*(*u*), and the amplitudes of the response curve are all within the saturation range. The simulations reveal that the output feedback control strategy with IS is effective.

**Fig 4:**
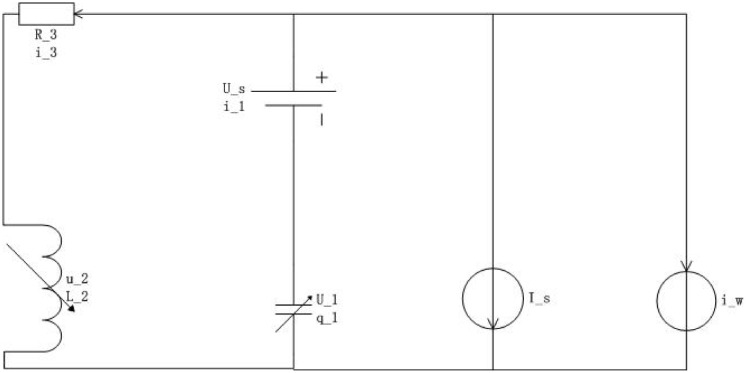
Nonlinear circuit system.

**Example 2.**Fig 4 ([18]) describes the nonlinear circuit system, where the magnetic flux *ψ* controls the inductance, the electric charge *q* controls the capacitance, *i*_3_ = *f*_1_(*ψ*) is current, *U*_1_ = *f*_2_(*q*) is voltage, and the current source disturbance is denoted by *i*_*w*_.

The system is written as follows by Kirchhoff’s Law,

$$\begin{array}{c} \left\{ \begin{array}{c} \dot{q}=-I_{s}-f_{1}\left(\psi \right)-i_{w},\\ \dot{\psi }=U_{s}+f_{2}\left(q\right)-R_{3}f_{1}\left(\psi \right). \end{array}\right. \end{array}$$ (42)

Denote $f_{1}\left(\psi \right)=\frac{4}{3}\psi^{\frac{1}{3}}$, $f_{2}\left(q\right)=\frac{4}{3}q^{\frac{1}{3}}$ and *R*_3_ = 3. Let the state *x* = (*x*_1_, *x*_2_)^T^ = (*q*, *ψ*)^T^, the control input *u* = (*u*_1_, *u*_2_)^T^ = (*I*_s_, *U*_s_)^T^, and the disturbance *d*(*t*) = *i*_*w*_. Then system (42) is expressed as the PCH system

$$\begin{array}{ccc} \dot{x} & = & \left(\begin{array}{c} \dot{q}\\ \dot{\psi } \end{array})=[(\begin{array}{cc} 0 & -1\\ 1 & 0 \end{array})-(\begin{array}{cc} 0 & 0\\ 0 & 3 \end{array})]\cdot (\begin{array}{c} \frac{4}{3}q^{\frac{1}{3}}\\ \frac{4}{3}\psi^{\frac{1}{3}} \end{array})+(\begin{array}{cc} -1 & 0\\ 0 & 1 \end{array})(\begin{array}{c} I_{s}\\ U_{s} \end{array}\right)\\ & & +(\begin{array}{c} -1\\ 0 \end{array})i_{w}≔[J(x)-R(x)]\nabla E\left(x\right)+g_{1}\left(x\right)u+g_{2}\left(x\right)d\left(t\right), \end{array}$$ (43)

where $E\left(x\right)=x_{1}^{\frac{4}{3}}+x_{2}^{\frac{4}{3}}$, $g_{1}\left(x\right)=(\begin{array}{cc} -1 & 0\\ 0 & 1 \end{array})$ and $g_{2}\left(x\right)=(\begin{array}{c} -1\\ 0 \end{array})$.

Considering IS, system (43) with disturbances is rewritten as

$$\begin{array}{c} \left\{ \begin{array}{c} \dot{x}=\left[J\left(x\right)-R\left(x\right)\right]\nabla E\left(x\right)+g_{1}\left(x\right)sat\left(u\right)+g_{2}\left(x\right)d\left(t\right),\\ y=g_{1}^{\text{T}}\left(x\right)\nabla E\left(x\right),\\ z=M\left(x\right)g_{1}^{\text{T}}\left(x\right)\nabla E\left(x\right), \end{array}\right. \end{array}$$ (44)

where *y*, *d*(*t*) and *z* are the output, the disturbance and the penalty signal, respectively. sat(u)∈R is the saturated control input and given as

$$\begin{array}{c} sat\left(u\right)=\{\begin{array}{c} 0.8,\;\;\;\;\;u>0.8,\\ u,\;\;\;\;\;0.8\geq u\geq -0.8,\\ -0.8,\;\;\;u<-0.8. \end{array} \end{array}$$ (45)

For disturbance attenuation level *γ* = 1, we choose symmetric matrix $K=(\begin{array}{cc} 3 & 0\\ 0 & 3 \end{array})$, the weighting matrix $M=(\begin{array}{cc} \frac{1}{2} & 0\\ 0 & \frac{1}{2} \end{array})$ and $∊=\frac{1}{30}$. Through verification, we find that these conditions in Theorem 3 hold, where

$$\begin{array}{c} \bar{K}=K+\frac{1}{2}M^{\text{T}}M+\frac{1}{2\gamma^{2}}I_{m}=(\begin{array}{cc} \frac{29}{8} & 0\\ 0 & \frac{29}{8} \end{array}), \end{array}$$ (46)

$$\begin{array}{c} \overset{ˇ}{R}\left(x\right)=R\left(x\right)+g_{1}\left(x\right)\left[K-\frac{1}{2}\epsilon {\bar{K}}^{\text{T}}\bar{K}-\frac{1}{2}I_{m}\right]g_{1}^{\text{T}}\left(x\right)=(\begin{array}{cc} 2.28 & 0\\ 0 & 5.28 \end{array})>0, \end{array}$$ (47)

$$\begin{array}{c} \tilde{R}\left(x\right)=\overset{ˇ}{R}\left(x\right)+\frac{1}{2\gamma^{2}}\left[g_{1}\left(x\right)g_{1}^{\text{T}}\left(x\right)-g_{2}\left(x\right)g_{2}^{\text{T}}\left(x\right)\right]=(\begin{array}{cc} 2.28 & 0\\ 0 & 5.78 \end{array})>0. \end{array}$$ (48)

According to Eq (29), the output feedback law is obtained

$$\begin{array}{c} u=-\bar{K}y=(\begin{array}{cc} \frac{29}{8} & 0\\ 0 & \frac{29}{8} \end{array})y. \end{array}$$ (49)

From Theorem 3, we know that controller (49) can effectively solve the problem of finite-time *H*_∞_ control of PCH system (44).

The simulation is carried out with initial condition *x*_1_(0) = −0.5 and *x*_2_(0) = 1.2. To verify the robustness of the proposed controller against disturbances, the current source disturbance *d*(*t*) = *i*_*w*_ = 1.1sin *t* is added into system (44) when 4*s* ≤ *t* ≤ 6*s*.

**Fig 5:**
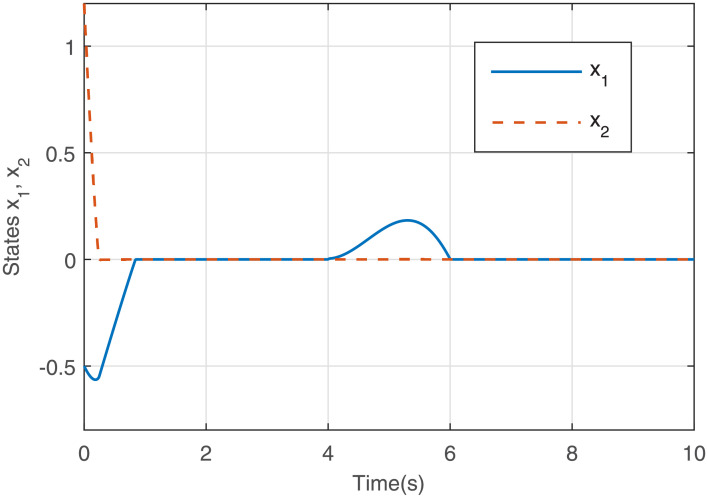
States *x*_1_ and *x*_2_ with disturbance and *sat*(*u*).

**Fig 6:**
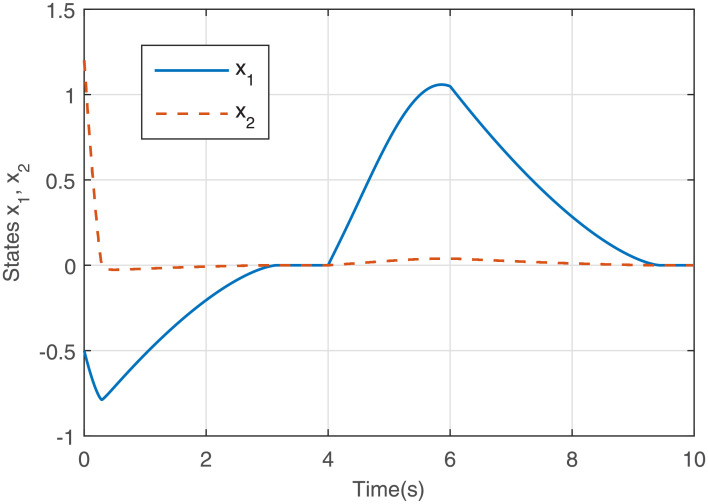
States *x*_1_ and *x*_2_ with disturbance and without *sat*(*u*).

**Fig 7:**
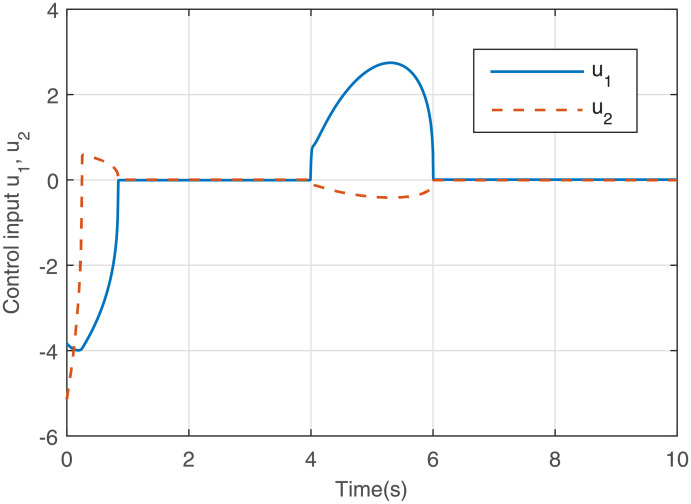
Response curves of the output feedback signal.

**Fig 8:**
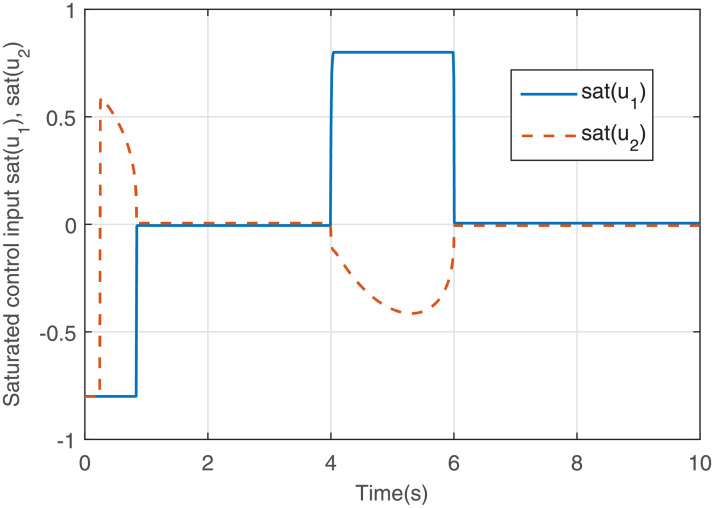
Response curves of the saturated control input *sat*(*u*).

Figs 5 to 8 give the simulation results. The response curves of states *x*_1_ and *x*_2_ with and without the saturated control input are shown in Figs 5 and 6, respectively. It can be seen that compared with Fig 6, the effect of the disturbance on the system is well suppressed when the disturbance appears in Fig 5. After the disturbance vanishes, the states converge to the equilibrium point faster in Fig 5. The response curves of the output feedback control are given in Fig 7. Fig 8 shows the response curves of the saturated control input *sat*(*u*), and the amplitudes of the response curves are all within the saturation range. The simulations illustrate that the output feedback controller is very effective against disturbances subject to IS. Therefore, when IS is considered, the proposed finite-time *H*_∞_ control strategy is valid.

## Conclusion

The problems of finite-time stabilization and finite-time *H*_∞_ control of PCH systems subject to disturbances and IS have been studied in this paper. By using system’s structure characteristics and an appropriate output feedback, the strictly dissipative PCH system has been obtained first. Second, the finite-time stabilization results for the case without and with IS have been presented using the Hamilton function method and truncation inequality technique. Next, the finite-time *H*_∞_ control problem for PCH system with disturbances and IS has also been solved. Finally, two examples have been proposed to illustrative the effectiveness of the theoretical results.

The control method proposed has been applied to two simulation examples in this paper. In fact, compared with the simulation results, the experimental results can better illustrate the effectiveness of the proposed method. It is of great theoretical and engineering significance to investigate engineering systems’ experiments using the proposed control scheme, which will be studied in the future.

## Supporting information

S1 File (ZIP)
