# Correction: Finite-time stabilization and *H*∞ control of Port-controlled Hamiltonian systems with disturbances and saturation

**DOI:** 10.1371/journal.pone.0272824

**Published:** 2022-08-04

**Authors:** Baozeng Fu, Qingzhi Wang, Ping Li

There is an error in affiliation 2 for authors Baozeng Fu and Qingzhi Wang. The correct affiliation 2 is: Shandong Key Laboratory of Industrial Control Technology, Qingdao, China.

*Laboratory for Experimental Biotechnology*, *Laboratory for Experimental Biotechnology,*
